# Secular Trends of Breast Cancer in China, South Korea, Japan and the United States: Application of the Age-Period-Cohort Analysis

**DOI:** 10.3390/ijerph121214993

**Published:** 2015-12-04

**Authors:** Zhenkun Wang, Junzhe Bao, Chuanhua Yu, Jinyao Wang, Chunhui Li

**Affiliations:** 1 Department of Epidemiology and Biostatistics, School of Public Health, Wuhan University, 115 Donghu Road, Wuhan 430071, China; wongzhenkun@gmail.com (Z.W.); junzhe_bao@126.com (J.B.); jinjinyao456@163.com (J.W.); 2 Global Health Institute, Wuhan University, 115 Donghu Road, Wuhan 430071, China; 3 School of Public Health, Dalian Medical University, 9 Westem Section, Lvshun South Street, Dlian 116044, China; chli0201@hotmail.com

**Keywords:** breast cancer, mortality, APC, East Asia, US

## Abstract

To describe the temporal trends of breast cancer mortality in East Asia and to better understand the causes of these trends, we analyzed the independent effects of chronological age, time period and birth cohort on breast cancer mortality trends using age-period-cohort (APC) analysis. We chose three main countries in East Asia, namely China, South Korea, and Japan, which have reported death status to the WHO Mortality Database, and used the United States as a comparison population. Our study shows that in general, breast cancer mortality rates in females increased in all three East Asian countries throughout the study period. By APC analysis, we confirmed that there is, in fact, a difference in age-specific mortality rate patterns between the Eastern and the Western countries, which is presumably caused by the two-disease model. While the cause of the decrease from approximately the 1950s generation is still in question, we believe that increasing general awareness and improvements in the health-care system have made a significant contribution to it. Although the age and cohort effects are relatively strong, the period effect may be a more critical factor in the mortality trend, mainly reflecting the increase in exposures to carcinogens and behavioral risk factors.

## 1. Introduction

Worldwide, breast cancer is the second most common type of cancer diagnosed in the general population and the most common among women. In 2012 alone, there were approximately 1.67 million new diagnosed cases, which accounted for a quarter of all new cancer cases [1]. Furthermore, it is the fifth leading cause of cancer death overall [2]. According to a report by Globocan, breast cancer, although still the second leading cause of cancer death in developed regions, is the most frequent cause of cancer death in women in less developed regions; this report shows 324,000 deaths in the developing world compared to 198,000 in the developed one (14.3% *vs*. 15.4%) [3]. 

The mortality rates from breast cancer in East Asian countries are relatively lower than in the West. However, this gap has been reduced due to a recently observed increasing secular trend of breast cancer mortality in East Asia, whereas mortality rates have been declining in Western countries for decades [4]. We do not know the cause of this difference due to the dearth of comparative research in the past.

Age, period, and cohort analysis (APC analysis) can be used to assess the character and quality of trends in the prevalence of key diseases, such as diabetes, cardiovascular disease and various types of cancers. This can be achieved by estimating the effects of these three time-dependent components on mortality rates separately, which allows the researcher to consider each component independently from the other two. In addition, it may be used to better depict the entire complex of social, historical, and environmental factors that simultaneously impact individuals and social groups [5]. Thus, using APC analysis to investigate secular trends in disease incidence and mortality rates could contribute certain clues to the etiology of the disease. APC analysis has already been successfully employed to assess mortality due to breast cancer in China [5,6], South Korea [7], Japan [8,9], and Hong Kong [10]. However, because these studies did not utilize exactly the same analytical models, their results are difficult to compare directly. To our best knowledge, no published research has focused on the comparison of breast cancer mortality trends in East Asia to the United States using APC analysis.

To describe the temporal trends of breast cancer mortality in East Asia and to better understand the causes of these trends, we analyzed the independent effects of chronological age, time period and birth cohort on mortality trends in breast cancer using APC analysis. We chose three main countries in East Asia, namely China, South Korea, and Japan, which have reported death status to the WHO Mortality Database, and used the United States as a comparison population. Due to the fact that the urban-rural difference of breast cancer in China has rarely been examined [11], China was further divided into two areas—rural and urban—in this study. We first described the trends in breast cancer mortality within the five areas. Then APC analysis was applied to determine if there were any differences in the effects of age, period, or cohort on these trends in mortality. Finally, possible causes of these trends were discussed in detail. It is our hope that studying the trends in breast cancer mortality within these geographical areas will not only lay a solid foundation for a better understanding of the relationship between social development and the cancer burden, but may also assist in exploring the influencing factors for breast cancer. The results of this study could serve as a scientific reference for the reduction of breast cancer mortality.

## 2. Materials and Methods 

The data cited on breast cancer deaths and population in this study are from the WHO Mortality Database and the Cancer Statistic Registries in the three main East Asian countries under study (China, South Korea, and Japan) [12]. To clarify, as explained above, China in this study refers to mainland China, which has been divided into urban and rural regions for the purpose of this study due to its large population and the healthcare gap between these areas [13]. For comparison, we also obtained corresponding data in the United States from the Surveillance, Epidemiology, and End Results (SEER) Database of the National Cancer Institute [14]. 

The focus of our study included breast cancer death cases diagnosed from 1953 to 2012; however, this data in South Korea and China were available only from 1985 to 1988 respectively. During the study period, the coding of the cause of death for breast cancer changed from the 7th to 10th revision of the International Classification of Disease (Code 170 in ICD-7, 174 in ICD-8 and ICD-9, C50 in ICD-10). However, fortunately, these changes have had little effect on the analysis of temporal trends for breast cancer [15]. Due to the fact that occurrence of breast cancer in those under 20 years old is very rare, and assessments of patients over 80 involve deaths from other competing causes, only rates for those within the age range of 20–79 were considered here [16,17]. 

To characterize the mortality trends, the mortality rate in five areas mentioned above was age-standardized by the world standard population proposed by Segi [18] and modified by Doll *et al*. [19]. These rates were smoothed with five-year moving averages which are commonly used with time series data to reduce short-term fluctuations and highlight longer-term trends or cycles. To perform APC analysis, the mortality and population data were arranged in five consecutive 5-year periods from 1988–1992 to 2008–2012 (for Japan and the U.S., a truncated calendar period from 1988 was employed to ensure the comparability of all areas) and twelve five-year age groups from 20–24 years to 75–79 years. The purpose of APC analysis, a statistical method commonly used in the fields of demography, sociology and epidemiology, is to evaluate the effects of age, period and cohort on demographic or disease rates. The age effects represent differing risks associated with various age groups; the period effects represent variations in vital rates over time that are associated with all age groups simultaneously; the cohort effects are associated with changes in rates across groups of individuals with the same birth years—that is, for successive age groups in successive time periods [20]. As there is a linear relationship between the age, period and cohort, it is difficult to estimate the unique set for every age, period and cohort effect, which is referred to as the non-identification problem [6]. To overcome this issue, we chose the Intrinsic Estimator (IE) method proposed by Fu due to its superior on estimation ability, non-bias, validity and asymptotic features [21]. The goodness-of-fit of the model was assessed by comparing the fit to the Akaike information criterion (AIC) and the Bayesian information criterion (BIC). All statistical analyses were carried out using the STATA 12 (Stata, College Station, TX, USA) and SAS 9.13 (SAS Institue, Cary, NC, USA) programs.

## 3. Results

**Figure 1:**
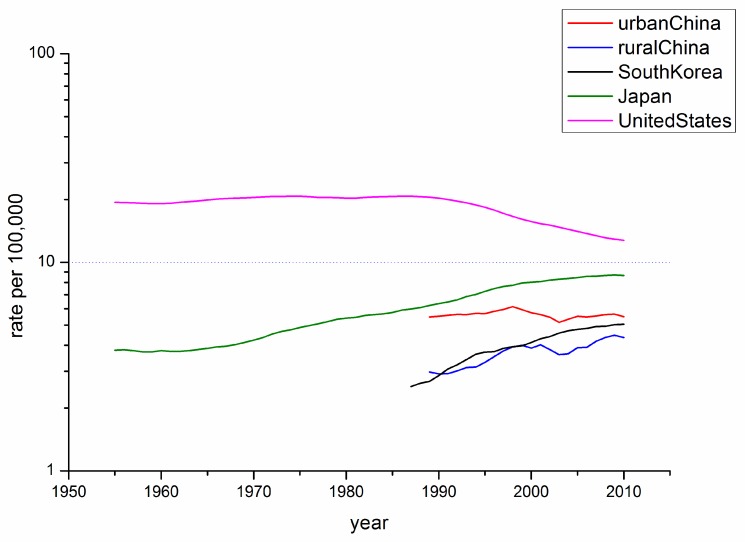
Trends in the five-year moving average world-standardized mortality rates per 100,000 population for breast cancer in the four East Asian areas and the US.

Age-specific mortality rates for breast cancer by year of death in the four countries are listed in Tables S1–S5. Figure 1 shows the trends of Age-Standardized Mortality Rate (ASMR) of breast cancer in China, South Korea, Japan and the U.S. for the study period of 1955–2010 (y-axis on a log scale), using five-year moving averages. The U.S. had the highest mortality rate, whereas the ASMRs of these the East Asian countries were below 10/100,000 throughout the observation period. The ASMR in the East Asian countries showed a general increase during the observation periods with the exception of urban China, which maintained a relatively stable trend. It must be noted that the ASMR in Japan leveled off until approximately 1965 before the constant incline. In contrast, the ASMR in the U.S. maintained its stability before 1990, which was followed by a significant decline.

**Figure 2:**
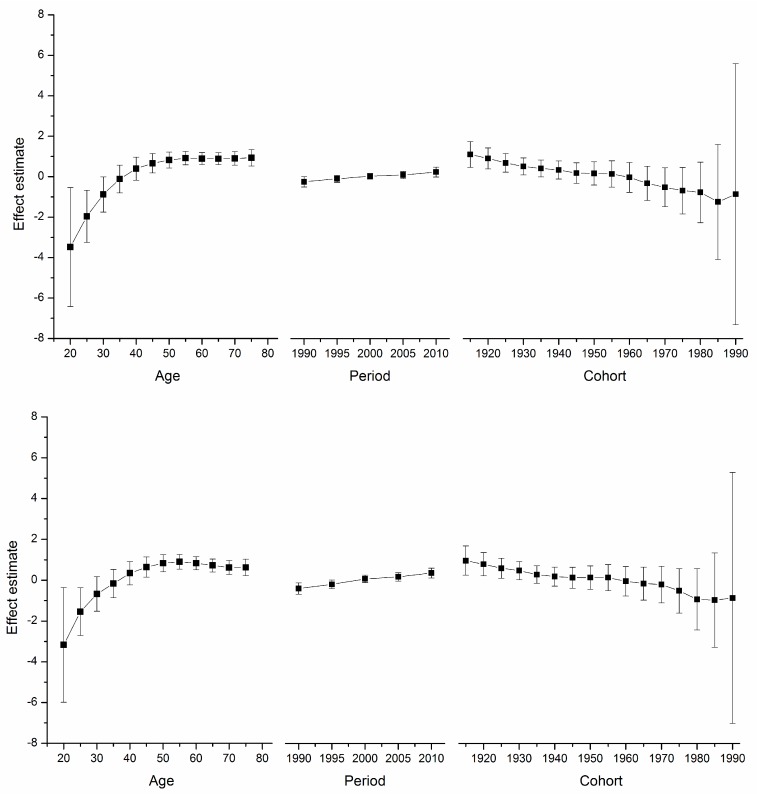
*Top*: Age, period and cohort effect on breast cancer mortality in urban China (bars around the point estimate indicate the 95% confidence intervals). *Bottom*: Age, period and cohort effect on breast cancer mortality in rural China (bars around the point estimate indicate the 95% confidence intervals).

**Figure 3:**
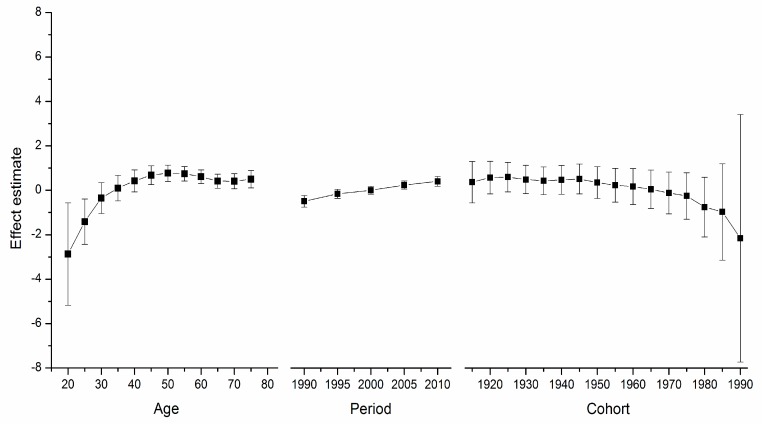
Age, period and cohort effect on breast cancer mortality in South Korea (bars around the point estimate indicate the 95% confidence intervals).

**Figure 4:**
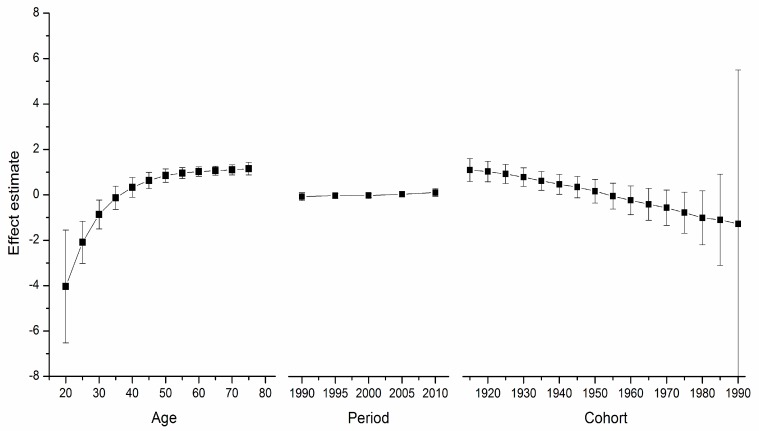
Age, period and cohort effect on breast cancer mortality in Japan (bars around the point estimate indicate the 95% confidence intervals).

**Figure 5:**
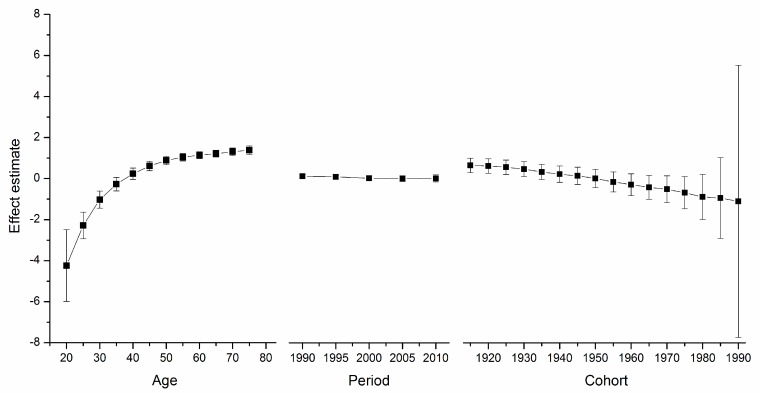
Age, period and cohort effect on breast cancer mortality in the US (bars around the point estimate indicate the 95% confidence intervals).

Table S6 depicts the goodness-of-fit for the APC models. These models, which utilize intrinsic estimator algorithm, were the best fitted to describe the trends in breast cancer mortality rate in the areas under study. Trends in age, period, and cohort effects in these four countries during the observation period are shown in Figure 2, Figure 3, Figure 4 and Figure 5. The specific effect results are displayed in Table S7. The Y-axis for each effect represents the natural logarithm of the relative mortality rate of breast cancer at the age, calendar year of death, or birth year of the patient listed. The characteristics of the trends in each were found to be as follows:

*Age* *Effect*. The age effect increased approximately linearly with increasing age from 20 to 54 in all areas. After 55 years of age, among women in the U.S., the age effect curve continued to rise at a slower pace, peaking near age 80. In contrast, age effects in East Asian areas either plateaued after age 55, such as in urban China and Japan, or began to decline in rural China and South Korea. Rural China’s age effect leveled off after age 70, and South Korea’s after age 65 but with a slight increase near age 80. 

*Period* *Effect*. The period effects in East Asian countries showed a trend of increase during the observation period, while there was a slight decline in the U.S. 

*Cohort* *Effect*. In all five areas, cohort effects were generally decreased. To be specific, urban China, rural China, South Korea, and Japan showed a constant decrease in the cohort effect from approximately the 1950s whereas that of the U.S. began earlier in the 1920s. The younger cohorts in the five areas showed a fluctuating curve because of the small number of observed deaths. In addition, different durations of stability were observed in these observation periods.

## 4. Discussion 

This is the first published study to use age, period, and cohort analysis to compare trends in breast cancer mortality across East Asia with those of the United States. This technique allowed estimations of when and how each of the three time-dependent parameters would affect these trends. The results of APC analysis may facilitate theorization regarding the etiology of the observed trends. However, we should not forget the affection of the “non-identification problem” associated within this technique. Many scholars have devised various solutions to overcome this vexing phenomenon [6]. In this study, the three variables were identified via the IE algorithm, which can be used to estimate linear and nonlinear components. This also facilitates identification of the model with the fewest assumptions possible imposed upon the data and on the linear effect of its shrinkage model. 

The ASMRs of breast cancer revealed contrary tendencies between the Eastern Asian countries and the U.S. during the observation period. Our results are consistent with the findings of a mortality trend discovered by Shin *et al*. [4], which uncovered secular trends in breast cancer mortality data in five Asian populations. Of note, the results of our APC analysis indicated that the mortality pattern of breast cancer in these areas can be predominately explained by the period effect, although the age and cohort effects were relatively strong. 

Our findings regarding the age effect of breast cancer mortality indicate an obvious difference between Eastern and Western women after menopause. Although this difference in age curves has been observed in many similar studies on breast cancer mortality and incidence, it was previously discounted due to uncontrolled cohort and period effects [22,23]. After adjusting these effects using the APC model, we confirmed that the difference in age-specific mortality rate patterns does, in fact, exist. This is consistent with Matsuno *et al.*’s research [24], which showed, via APC analysis, that the difference in age-specific incidence rate patterns persists. 

For the women in the U.S., the age effect was found to increase exponentially until age 50, when it continues to rise, albeit at a slower pace. This phenomenon, which has been observed in several Western countries and it affects both incidence and mortality, is known as the “Clemmensen hook” [25,26]. The hypothesis presented here involves a two-disease model of breast cancer, which can be visualized as two overlapping curves representing tumors in pre- and post-menopausal women, respectively [27]. Figure 2, Figure 3, Figure 4 and Figure 5 show that the age effect distribution of breast cancer deaths before menopause in East Asian countries was consistent with that in U.S.; however, they changed significantly after menopause. It is worth mentioning that, in the same study [24], Matsuno *et al.* suggests that the U.S. population suffers bimodal (early- and late- onset) breast cancer incidences, while Japan only experiences an early-onset age distribution. We propose that the two-disease model should be the key to these differences, and the reason for the large difference in postmenopausal disease components is probably not due to racial differences [28]. One possible explanation is that nutrition, life-style, and the high prevalence of obesity in Western nations serves to explain the higher level of breast cancer as due to the higher rate of incidence of postmenopausal disease [29], which is supported by migrant studies [30]. According to a study of Japanese immigrants [24], the U.S. age effect distribution of breast cancer was also observed in Japanese-Americans in Hawaii. American women and those in other Western countries could benefit from East Asian studies that identify the probable influencing factors.

The cohort effects of the very young and very old patients must be interpreted carefully because of the small number of observations upon which they are based; they have larger standard errors than estimates for the middle cohorts [21]. Therefore, we focused on the general trends of cohort effect in the middle range. Overall, risk by birth cohort showed a downward trend except some periods which level off or slightly increase. Of note, all four East Asian areas witnessed a consistent decline from the 1950’s generation, while U.S. experienced a similar trend in the 1920s. These findings are similar to observations in other studies on breast cancer mortality in China [5], Japan [8,9], South Korea [7] and many Western countries including the U.S [15,31]. However, these declines of cohort effect were somehow contradictory to expectation because these they usually mirror trends in risk factors, and trends in most known and suspected risk factors would indicate an incline in risk of breast cancer due to the cohort effect.

Risk factors related to cohorts mainly include some reproductive factors (early menarche, late menopause, child bearing patterns, and decreased breast-feeding time/proportion), life-style (increased use of alcohol and/or cigarettes) and dietary habits (increased consumption of a Western-style diet, including high intake of dietary fat and/or calories). Obviously, these factors cannot be the driving forcing of the declines of birth effects except that there is another view on child bearing patterns. According to a study on breast cancer mortality in 20 Western countries [31], the downward trend from approximately the 1920 to 1945 birth cohort for most of these countries, including US, may be partly due to child bearing patterns since there has been a general reduction in the percentage of childless women by age 40 and mean age at first birth between the 1930 and 1945 birth cohorts. It seems that there are similar reasons for Eastern Asian countries due to the baby booms after wars (World War II, China’s War of Liberation, and the Korean War). But we should note that the proportion of nulliparous women in the U.S. increased after the 1940s cohort [32] and the baby boom did not persist in East Asian countries. It is highly unlikely that changes in reproductive behavior are the sole cause of these monotonic declines in the birth effects.

While there is still some question regarding the causes of the decrease in recent birth cohorts, improvements in health care—both in treatment and accessibility—and increasing awareness of breast cancer among women are likely. Increased education of females and their participation in the labor force may have the effect of delaying childbearing and reducing the number of children; however, these factors have also raised women’s awareness of critical health issues and improved their socioeconomic status, as observed in more recent female cohorts [15]. Greater access to health insurance and medical care also has reduced the risk of morbidity and mortality [15]. These findings may indicate that early access to and accumulation of these resources has had pronounced positive effects upon survival of breast cancer.

Here, period effects were found to be small or modest when birth cohort and age effects were both controlled. From Figure 2, Figure 3, Figure 4 and Figure 5, we cannot identify the role of period effect due to that the study period (1988–2012) was relatively short and the trends of period effect were increased or decreased monotonically. For these reasons, we carried out another APC-IE analysis on U.S. and Japanese population using their relatively long-term data (1953–2012), the results of which are shown in Figure S1. We found that the period effect might be a more critical factor in the trend of breast cancer mortality than the other two effects. This is because, according to Figure 1 and Figure S1, the period effect in Japan began to increase from approximately 1965 and its mortality rate inclined simultaneously, while the period effect in the U.S. decreased from approximately 1990 along with its mortality rate. However, this correlation needs to be further verified due to insufficiency of relevant data in China and South Korea. 

Moreover, our results (see Figure 2, Figure 3, Figure 4 and Figure 5) showed that period effects in all four East Asian areas increase throughout the study period. It is easy to raise questions regarding changes in death certification; however, the diagnosis of terminal breast cancer is usually relatively clear-cut, so the results here reported for East Asia are unlikely to be attributable to improvements in detection [33]. 

Generally, trends observed over a calendar year may indicate: (a) the impact of novel medical interventions, such as, improved screening or/and treatment, better access to healthcare and the patients’ response to the interventions; and (b) reductions in exposure to carcinogens or changes in behavioral risk factors [20]. Specifically, changes in nutrient intake may have contributed to both the cohort and period effect. Widespread changes in diet after World War II, involving both increased caloric intake and proportions of fat, according to Minami *et al*. [9], may have increased the number of obese women, who may be more susceptible to postmenopausal breast cancer. Although it was also not possible to obtain detailed data regarding the relationship between dietary intake and BMI in this study, these changes in diet may be likely to have contributed to the period effect. At the same time, we cannot ignore the impact of novel medical interventions. For example, the sudden decline in period effect and breast-cancer mortality in the U.S. in the early 1990s was suggested to probably be largely attributed to adjuvant systemic therapy and the earlier detection of palpable tumors [31,34]. We believe that better access to healthcare access may partially explain the slightly slower rise of the period effect in urban China in comparison to rural China. 

Our study has some specific limitations. First of all, the data from the WHO Mortality Database were supplied by the governments of the various countries and districts, which may have substantially different systems for collecting vital statistics and methods used to confirm causes of death. These factors limit the comparability of the information in these databases. Large swaths of China and the United States are not covered by cancer registries, which may also limit the representativeness of the results. However, it is here assumed that the database is reliable enough for the purposes of this study, which is the interpretation of general trends in the areas considered here. Second, the data obtained from China and South Korea was not sufficient to monitor the significant influence of period effect to mortality trend due to the relatively short observation time. A longer period of observation in the future and further studies are still needed. Third, the research objectives in this study did not cover all the East Asian countries because data in North Korea and Mongolia were not available in the WHO Mortality Database. However, fortunately, China, Japan and South Korea cover the majority of the East Asian population, so we believe they are good representatives of the East Asia.

## 5. Conclusions 

In summary, our study shows that in general, breast cancer mortality rate in females increased in all three East Asian countries throughout their study period respectively except Japan have a stable time before 1965. By APC analysis, we confirmed that there is, in fact, a difference in age-specific mortality rate patterns between the Eastern and the Western countries, which is presumably caused by the two-disease model. While the cause of the decrease from approximately the 1950’s generation is still in question, we believe that increasing general awareness and improvements in the health-care system have made a significant contribution to it. Although the age and cohort effects are relatively strong, the period effect may be a more critical factor in the mortality trend, mainly reflecting the increase in exposures to carcinogens and behavioral risk factors.

## Supplementary Files

Supplementary File 1
