# Supplementary material for: Secular Trends of Breast Cancer in China, South Korea, Japan and the United States: Application of the Age-Period-Cohort Analysis
Source: Int J Environ Res Public Health. 2015 Dec 4;12(12):15409–18. doi: 10.3390/ijerph121214993 (PMC4690929; doi:10.3390/ijerph121214993)
Supplement: Supplementary File 1 [file ijerph-12-14993-s001.pdf]

Secular Trends of Breast Cancer in China, South Korea, Japan and the United States: Application of the Age-Period-Cohort Analysis

Table S1. Age-specific mortality rate for breast cancer by year of death in urban China (10,000).

Age	Year of Death				
	1988–1992	1993–1997	1998–2002	2003–2007	2008–2012
20–24	0.12	0.10	0.06	0.04	0.10
25–29	0.53	0.40	0.36	0.50	0.33
30–34	2.14	1.74	1.21	1.44	1.43
35–39	4.94	4.84	3.39	3.93	3.45
40–44	8.04	9.96	8.50	7.35	6.73
45–49	10.79	12.58	12.88	13.19	10.96
50–54	15.20	14.35	16.70	18.49	16.35
55–59	17.33	18.07	20.78	19.92	21.01
60–64	19.30	20.18	22.68	18.48	21.85
65–69	22.79	23.07	22.80	22.02	22.07
70–74	29.28	27.46	25.57	23.78	26.64
75–79	36.26	34.34	30.92	27.07	31.32

Table S2. Age-specific mortality rate for breast cancer by year of death in rural China (10,000).

Age	Year of Death				
	1988–1992	1993–1997	1998–2002	2003–2007	2008–2012
20–24	0.09	0.12	0.09	0.05	0.11
25–29	0.44	0.53	0.67	0.52	0.54
30–34	1.38	1.63	2.09	1.52	1.16
35–39	2.72	3.22	3.27	3.56	3.12
40–44	4.69	6.12	5.82	6.50	7.14
45–49	6.68	8.37	10.03	8.80	10.10
50–54	8.03	9.57	12.03	13.07	14.60
55–59	9.64	10.73	12.19	15.02	18.34
60–64	10.16	11.02	13.66	13.99	15.98
65–69	10.38	10.94	14.85	12.46	14.67
70–74	13.38	12.89	13.67	11.20	13.78
75–79	14.48	13.43	15.74	17.77	15.02

Table S3. Age-specific mortality rate for breast cancer by year of death in South Korea (10,000).

Age	Year of Death				
	**1988–1992**	**1993–1997**	**1998–2002**	**2003–2007**	**2008–2012**
20–24	0.10	0.20	0.11	0.12	0.04
25–29	0.70	0.88	0.81	0.49	0.59
30–34	2.11	2.73	2.58	2.92	2.25
35–39	3.42	4.75	5.07	5.36	5.48
40–44	5.86	6.95	7.50	8.82	8.63
45–49	9.45	9.62	10.87	12.52	12.86
50–54	9.70	12.07	13.13	14.45	16.73
55–59	8.53	11.80	14.81	16.38	17.15
60–64	7.43	10.58	12.46	16.83	16.81
65–69	6.59	9.65	9.58	13.32	16.13
70–74	6.28	10.31	10.95	12.83	15.09
75–79	6.18	11.30	12.98	13.73	15.96

Table S4. Age-specific mortality rate for breast cancer by year of death in Japan (10,000).

Age	Year of Death											
	1953–1957	1958–1962	1963–1967	1968–1972	1973–1977	1978–1982	1983–1987	1988–1992	1993–1997	1998–2002	2003–2007	2008–2012
20–24	0.04	0.07	0.06	0.08	0.12	0.07	0.14	0.06	0.10	0.07	0.09	0.05
25–29	0.46	0.44	0.46	0.47	0.61	0.83	0.73	0.66	0.51	0.55	0.52	0.47
30–34	1.73	1.68	1.75	2.12	2.42	2.63	2.77	2.79	2.51	2.70	2.26	1.91
35–39	3.97	3.93	4.07	4.31	4.70	5.05	5.56	6.51	6.82	6.64	5.78	4.97
40–44	6.91	6.65	7.05	7.14	8.24	8.56	9.25	10.94	12.16	12.09	11.47	10.67
45–49	8.53	9.62	9.82	10.85	12.36	12.92	13.90	15.24	18.55	20.39	19.50	17.92
50–54	10.30	10.91	12.11	13.56	16.11	17.24	17.98	20.43	24.21	27.94	29.41	27.24
55–59	11.84	11.56	12.46	14.20	16.37	19.99	20.69	22.85	25.88	29.55	34.24	36.11
60–64	12.51	11.50	11.77	13.65	15.87	18.40	20.65	21.57	24.55	28.24	31.69	36.16
65–69	13.40	12.46	12.01	12.86	15.47	17.94	18.60	20.43	24.15	26.29	29.70	33.75
70–74	15.32	14.71	13.30	13.49	14.61	17.34	18.19	20.34	22.58	25.61	27.95	32.52
75–79	17.49	15.93	15.08	16.05	16.81	17.18	18.09	19.84	23.51	26.45	29.68	33.11

Table S5. Age-specific mortality rate for breast cancer by year of death in US (10,000).

Age	Year of Death											
	1953–1957	1958–1962	1963–1967	1968–1972	1973–1977	1978–1982	1983–1987	1988–1992	1993–1997	1998–2002	2003–2007	2008–2012
20–24	0.24	0.21	0.28	0.20	0.21	0.15	0.15	0.10	0.14	0.08	0.12	0.08
25–29	1.61	1.53	1.65	1.80	1.48	1.28	1.22	1.19	1.01	0.83	0.69	0.66
30–34	5.86	6.03	5.86	5.72	5.58	5.38	5.10	4.59	3.94	3.64	3.04	2.60
35–39	13.78	13.72	13.59	12.88	12.54	12.29	12.90	11.76	10.07	8.60	7.65	6.68
40–44	26.87	25.97	26.08	26.25	23.74	23.05	23.02	22.52	19.41	16.14	14.21	13.25
45–49	44.18	41.51	42.72	43.61	42.07	37.79	36.61	36.45	32.86	26.25	22.97	20.86
50–54	53.79	54.60	59.95	60.22	59.66	57.31	54.16	52.55	48.16	40.11	34.20	31.14
55–59	61.94	63.71	66.08	72.47	74.16	73.34	72.56	68.88	60.97	53.00	47.80	41.38
60–64	69.97	70.51	78.73	78.62	84.02	84.47	89.69	85.43	74.91	65.08	61.14	55.01
65–69	80.71	81.83	83.08	89.28	91.18	94.76	99.99	100.86	91.38	78.02	71.38	68.99
70–74	98.61	95.60	94.16	98.01	108.44	104.97	113.75	117.26	110.47	96.92	85.78	80.55
75–79	123.01	109.04	107.49	111.65	113.78	117.75	123.04	132.51	129.60	116.16	107.46	98.29

Table S6. Summary statistics comparing goodness-of-fit for different models.

		Age Only	Age + Period	Age + Cohort	APC (IE)
urban China	AIC	839.6	341.9	427.4	5.0
	BIC	920.1	396.7	459.9	−131.3
rural China	AIC	746.4	305.4	395.1	4.6
	BIC	819.3	339.3	458.0	−126.7
South Korea	AIC	1025.3	524.1	496.6	4.6
	BIC	1134.2	576.8	603.4	−122.1
Japan	AIC	868.0	432.7	1162.6	5.0
	BIC	986.3	484.5	1616.7	−122.6
United States	AIC	960.2	375.8	456.9	5.7
	BIC	1176.3	408.0	482.4	−122.7

Table S7. APC model analysis results of breast cancer mortality.

	Urban China		Rural China		South Korea		Japan		US	
	Coef.	SE	Coef.	SE	Coef.	SE	Coef.	SE	Coef.	SE
**Age (year)**										
20–24	−3.48	1.50	−3.17	1.44	−2.87	1.18	−3.60	2.95	−4.24	1.75
25–29	−1.96	0.66	−1.54	0.60	−1.42	0.52	−1.90	1.17	−2.29	0.65
30–34	−0.88	0.44	−0.67	0.43	−0.35	0.35	−0.64	0.77	−1.03	0.42
35–39	−0.11	0.35	−0.16	0.35	0.10	0.29	0.03	0.62	−0.27	0.33
40–44	0.40	0.29	0.34	0.29	0.42	0.25	0.44	0.52	0.24	0.28
45–49	0.66	0.24	0.64	0.25	0.68	0.22	0.74	0.44	0.61	0.23
50–54	0.83	0.20	0.84	0.21	0.77	0.19	0.95	0.36	0.88	0.20
55–59	0.92	0.17	0.91	0.18	0.74	0.17	0.99	0.30	1.04	0.18
60–64	0.90	0.15	0.83	0.17	0.61	0.16	0.88	0.27	1.14	0.17
65–69	0.89	0.15	0.73	0.16	0.41	0.16	0.78	0.26	1.21	0.17
70–74	0.91	0.17	0.62	0.17	0.41	0.17	0.68	0.29	1.31	0.18
75–79	0.94	0.21	0.63	0.20	0.49	0.20	0.65	0.35	1.39	0.20
**Period (year)**										
1990	−0.25	0.13	−0.40	0.14	−0.49	0.13	−0.38	0.23	0.06	0.08
1995	−0.10	0.09	−0.20	0.11	−0.16	0.10	−0.17	0.16	0.06	0.08
2000	0.03	0.07	0.07	0.09	0.00	0.09	0.02	0.13	0.02	0.11
2005	0.09	0.09	0.17	0.10	0.24	0.10	0.18	0.15	0.03	0.14
2010	0.23	0.12	0.36	0.12	0.41	0.11	0.35	0.21	0.07	0.17
**Cohort (year)**										
1915–1919	1.10	0.32	0.97	0.36	0.37	0.48	0.77	0.73	0.65	0.35
1920–1924	0.91	0.27	0.79	0.29	0.57	0.37	0.75	0.63	0.61	0.35
1925–1929	0.68	0.23	0.59	0.25	0.60	0.34	0.67	0.58	0.56	0.35
1930–1934	0.51	0.22	0.48	0.23	0.49	0.33	0.62	0.56	0.46	0.36
1935–1939	0.41	0.21	0.28	0.22	0.43	0.32	0.54	0.56	0.33	0.37
1940–1944	0.34	0.23	0.18	0.24	0.47	0.33	0.49	0.58	0.22	0.40
1945–1949	0.18	0.26	0.13	0.26	0.51	0.34	0.44	0.62	0.14	0.42
1950–1954	0.16	0.29	0.14	0.29	0.35	0.36	0.41	0.67	0.01	0.45
1955–1959	0.14	0.33	0.14	0.33	0.23	0.39	0.30	0.73	−0.16	0.49
1960–1964	−0.03	0.38	−0.05	0.37	0.17	0.41	0.08	0.79	−0.29	0.53

Table S7. *Cont.*

	Urban China		Rural China		South Korea		Japan		US	
**Cohort (year)**	Coef.	SE	Coef.	SE	Coef.	SE	Coef.	SE	Coef.	SE
1965–1969	−0.32	0.43	−0.16	0.41	0.05	0.44	−0.14	0.87	−0.42	0.58
1970–1974	−0.52	0.49	−0.21	0.46	−0.12	0.48	−0.38	0.96	−0.52	0.65
1975–1979	−0.69	0.59	−0.52	0.56	−0.25	0.53	−0.70	1.11	−0.69	0.78
1980–1984	−0.77	0.76	−0.93	0.77	−0.75	0.69	−0.98	1.43	−0.89	1.10
1985–1989	−1.24	1.45	−0.97	1.18	−0.97	1.10	−1.12	2.40	−0.95	1.96
1990–1994	−0.86	3.29	−0.87	3.14	−2.15	4.68	−1.73	8.81	−1.11	6.63

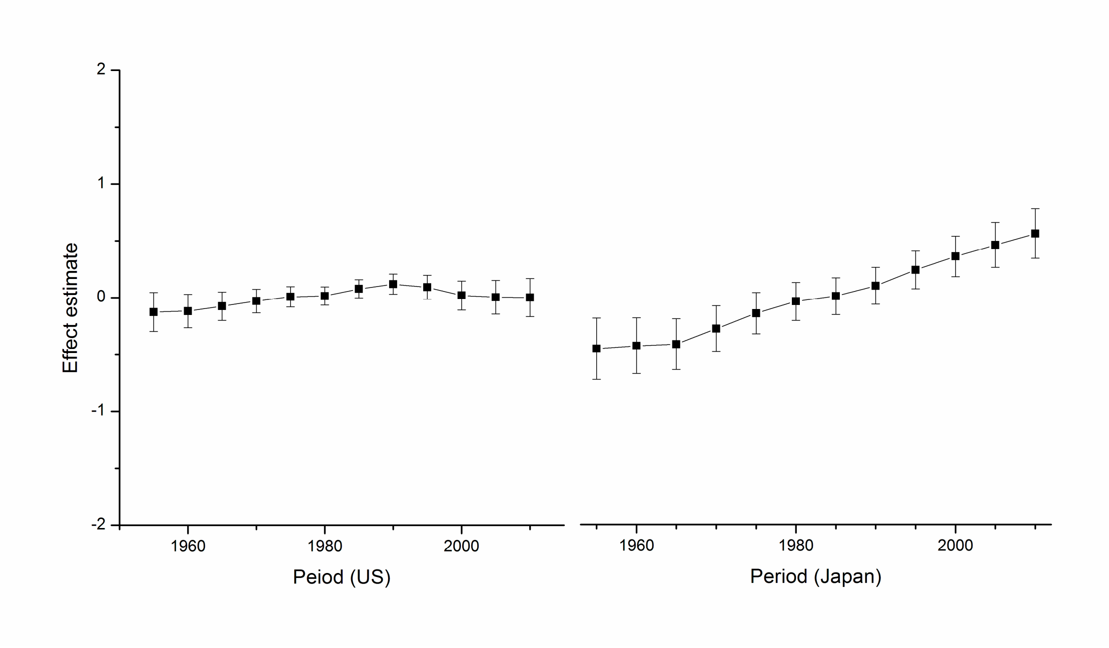

Figure S1. Period effect on breast cancer mortality from the results of APC-IE analysis in the US and Japan (bars around the point estimate indicate the 95% confidence intervals).

© 2015 by the authors; licensee MDPI, Basel, Switzerland. This article is an open access article distributed under the terms and conditions of the Creative Commons Attribution license (http://creativecommons.org/licenses/by/4.0/).
